# Resistance training modifies of serum levels of matrix metalloproteinase 2 and tissue inhibitor of matrix metalloproteinases in multiple sclerosis women - a randomized controlled trail

**DOI:** 10.1186/s12868-024-00856-1

**Published:** 2024-03-04

**Authors:** Nasrin Niazi Nezhad, Abdolhossein Parnow, Kianoosh Khamoushian, Rasoul Eslami, Julien S Baker

**Affiliations:** 1 Sport Bio-Sciences Department, Physical Education and Sports Sciences Faculty, Razi University, University Street, Kermanshah, Iran; 2 Department of Neurology, Kermanshah University of Medical Sciences, Kermanshah, Iran; 3 Exercise Physiology Department, Faculty of Physical Education and Sport Sciences, Allameh Tabataba’i University, Tehran, Iran; 4 Institute for Population Health and Medical Informatics, Hong Kong Baptist University, Kowloon, Hong Kong China

**Keywords:** Blood-brain barrier, Matrix metalloproteinases, Multiple sclerosis, Resistance training

## Abstract

The objectives of the present study was to investigate the effects of resistance training (RT) on serum levels of controlling blood-brain barrier (BBB) permeability indices and cognitive performance in MS women (MS-W). In this randomized control trail study (IRCT registration code: IRCT20120912010824N3, 07.09.2023), twenty-five MS-W were randomly divided into sedentary (MS) and resistance exercise (12 weeks/3 times per week/ 60–80% of 1RM) (MS + RT) groups. Fifteen healthy aged-matched women participated as a control group (HCON). The serum level of matrix metalloproteinase-2 (MMP-2), matrix metallopeptidase-9 (MMP-9), tissue metalloproteinase inhibitors-1 (TIMP-1), tissue metalloproteinase inhibitors-2 (TIMP-2), and S100 calcium-binding protein B (S100B) were assessed. In addition, cognitive performance was assessed pre- and post- intervention with the Brief International Cognitive Assessment for MS (BICAMS). A significant reduction in MMP-2, TIMP-2 serum levels, and MMP-2/TIMP-2 ratio were observed in post-test for MS + RT group (*p* < 0.01) in comparison to the HCON and MS groups; however, no changes were observed in MMP-9, TIMP-1, S100B and MMP-9/TIMP-1 ratio after RT (*p* > 0.05). The verbal learning was improved in post-test for MS + RT group (*p* < 0.01), although no change were observed for visuospatial memory and information processing speed (*p* > 0.05). These findings suggest that resistance training can modify some indices of BBB permeability and improve verbal learning in MS-W. The findings may also be beneficial as a non-pharmacological intervention to reduce inflammation.

## Introduction

Multiple sclerosis (MS) is a chronic inflammatory and neurological disease of the central nervous system (CNS) that its prevalence in women is three times higher than in men indicating which sex-related factors have an effect on an individual’s susceptibility to developing the condition [1, 2]. MS occurs when lymphocytes cross the blood-brain barrier (BBB) and initiate axonal demyelinating processes [1]. Alterations in the functioning of the BBB, particularly increase resulting from permeability, is major problem for patients with MS [3–5]. In other words, entering of leukocytes to the CNS during periods of BBB disruption initiates a cascade of events that leads to demyelination and axonal loss and eventually progressive disability [4].

Many signaling pathways, including matrix metalloproteinases (MMPs), have been identified to control BBB permeability through the regulation of structural components inculding degrade and remodel the proteins that form the ECM (collagen IV, V, laminin, fibronectin and proteins creating tight junction in endothelium of BBB: ZO-1 and occludins) [5, 6]. MMPs play a key role in the function of the BBB and the pathogenesis of MS [7]. MMPs such as MMP-2 and MMP-9 are expressed by neurons, astrocytes, and microglia in the CNS, and they play a physiopathological role in the disruption of the BBB through digest the endothelial basal lamina, microglia activation and further progression of inflammatory cells [3, 7]. In addation, MMPs regulate several functions related to inflammation including bioavailability and activity of inflammatory cytokines and chemokines [8].

Studies have claimed that matrix MMP-2 and MMP-9 are markers of BBB disruption [1, 3, 7, 9]. Clinical studies have demonstrated that serum MMP-2 and MMP-9 were elevated in different MS subtypes [10–13] and this increase related with BBB leakage [5]. As the proteolytic activity of MMPs is regulated by tissue inhibitors of MMPs (TIMPs), the imbalance of MMPs and TIMPs cause a series of effects that cause BBB breakdown and the infiltration of peripheral blood leukocytes to CNS [7].

Calcium-binding protein B (S100B) is a small Ca2 + and Zn2+-binding protein mainly expressed by astrocytes and a small subset of oligodendrocytes [5, 14, 15] and gets extravasation into the bloodstream only when the BBB is disrupted [5]. In MS patients, increased levels of S100B in CSF and serum have been reported, and this increase may cause activation of microglia and astrocytes, as well as oligodendrocyte demise, exacerbating tissue damage during an MS episode or delaying re-myelination [5, 15]. Interestingly, the elevation of the inflammatory molecule S100B in the serum of MS patients has been associated with BBB disruption [5, 15].

Recently, exercise training has been considered an important non-pharmacological treatment method for reducing symptoms and side effects of MS [16, 17]. However, scientific evidence relating to exercise effects on MS pathophysiology including BBB function is limited. Few studies have investigated the effects of regular exercise on BBB parameters in MS patients [16, 18], although exercise-induced alterations in levels of MMP-9 [9, 19] and MMP-2 [9] have been observed. These changes are important in the function of the BBB in MS patients. Although previous studies have investigated the effects of exercise on serum levels of MMPs, its possible effect on the serum concentrations of tissue-specific inhibitors MMPs (TIMP-1 and TIMP-2) and MMP/TIMP homeostasis in MS patients remains unclear.

Since the MMPs/TIMPs ratio seems to provide a better measurement of proteolytic activity, and the effectiveness of resistance training in modulating BBB permeability in MS patients is unknown as well as the widespread prevalence of this disease in women compared to men; we hypothesized that a 12-week resistance training (RT) has effect on serum levels of S100B, MMP-2, 9, TIMP-1, 2, and cognitive performance with MS women (MS-W).

### Materials and methods

#### Research design and participants

This study is a RCT (parallel-group) using a quasi-experimental design with pre- and post-testing which was performed in accordance with the Declaration of Helsinki and approved by the Ethics Committee of the Ethics in Research Working Group at the Sports Sciences Research Institute (ethical code: SSRI.REC-1402-101; IRCT registration code: IRCT20120912010824N3, 07.09.2023), Iran. “Informed” consent was obtained from all participants. The full trial was conducted from July, 2021 to February, 2022.

**Fig. 1:**
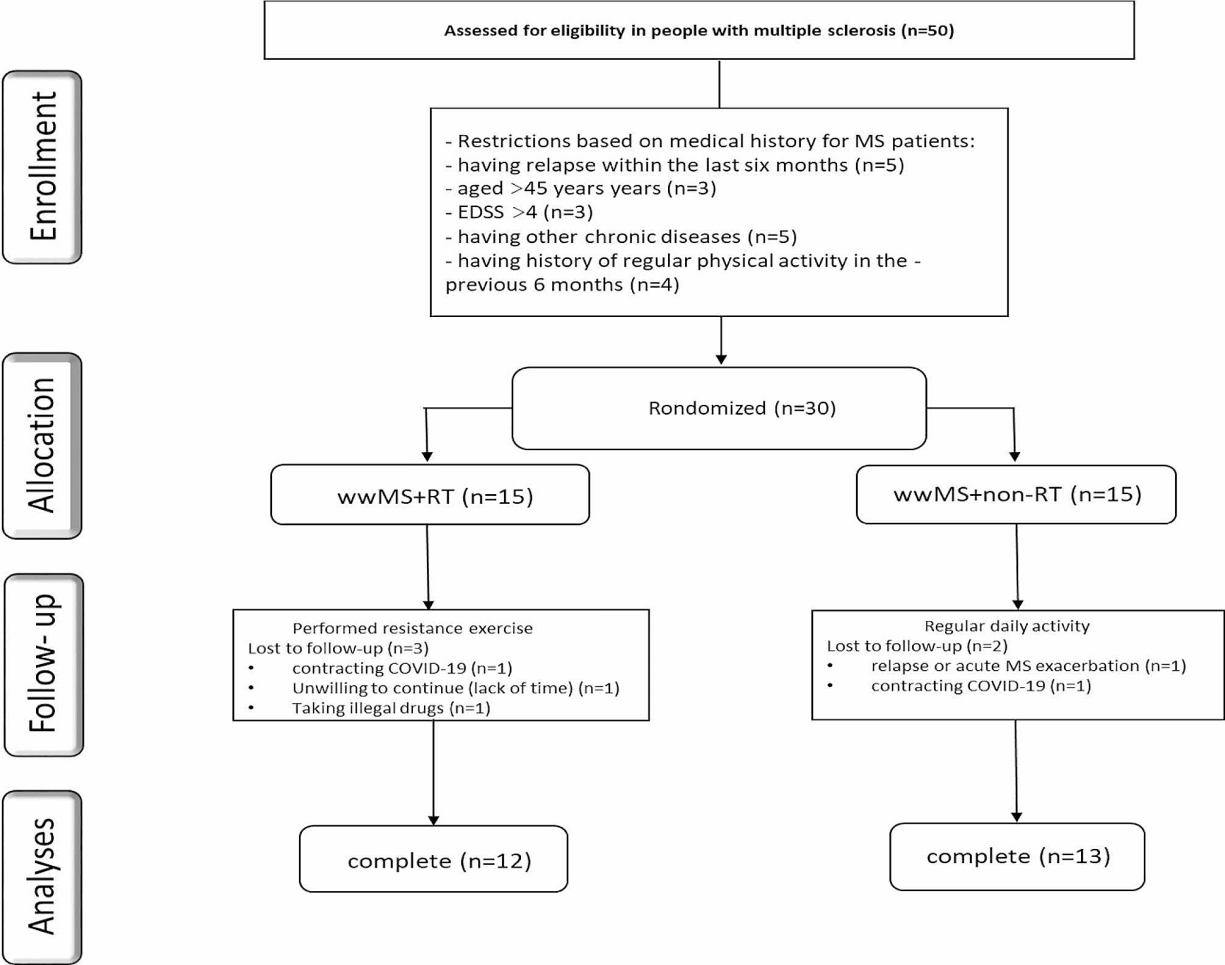
Participant recruitment flow chart HCON: Health group; MS: Multiple Sclerosis; MS + RT: Multiple Sclerosis + resistance training group

Women with MS (relapsing-remitting MS (RRMS)) were recruited through advertising at the Kermanshah multiple sclerosis center, Iran. Fifty MS-W (RRMS) volunteered to participate in the current study. Subsequently, thirty eligible MS-W (RRMS) were considered to enter the RCT based on inclusion/exclusion criteria (Fig. 1). The inclusion criteria were as follows: (a) history of at least two-year diagnosed MS; (b) no relapse or acute MS exacerbation within the last six months; (c) aged between 18 and 45 years; (d) EDSS ≤ 4; (e) no other chronic diseases (metabolic, cardiovascular, renal,…); (f) no history of regular physical activity in the previous 6 months. Exclusion criteria comprised: (a) severe relapses during the study period; (b) participation in any extra exercise training programs; (c) smoking and consuming other drugs (except MS medications); (d) lack of regular attendance in the intervention; (e) COVID-19 infection. To estimate the sample size in the present study, G*Power software [14] was used based on statistical power of 80% with a significance level of 0.5, effect size of 6.0, and standard deviation (SD) 0.5 based on previous studies. A sample size of 13 achieved with an estimated drop-out of 15% we included 15 participants per group.

Finally, participants were assigned in two (a) MS (*n* = 15) and (b) MS + resistance training (MS + RT) (*n* = 15) groups with using a random number table by sport specialist. In addition, the fifteen healthy and non-exercised women were selected as healthy control group (HCON; *n* = 15) to monitored current research data. Participants in MS and HCON groups were instructed to keep their daily activity during the trial period without any exercise training; indeed, MS + RT group performed RT program based on Table 1. MS-W groups (with or without RT) received their drug treatment supervised by specialist neurologist. It should be mentioned here that 5 participants (Three and two people from MS + RT and MS groups, respectively) were excluded from the study due to contracting COVID-19, relapse, unwillingness to continue cooperation, and taking illegal drugs.

**Table 1:** Resistance training (RT) program

Weeks	Load (%1RM)	Rep*Sets	Rest (minutes)
1	60	10*3	**2**
2	60	12*3	**2**
3	70	8*3	**2**
4	70	10*3	**2**
5 (1st session)	1RM Assessment		
5 (2nd and 3rd sessions)	75	8*3	**2**
6	75	8*4	**2**
7	65	10*4	**2**
8	65	12*4	**2**
9 (1st session)	1RM Assessment		
9 (2nd and 3rd sessions)	75	8*4	**2**
10	75	10*4	**2**
11	80	5*4	**3**
12	80	6 *4	**3**

Rep: A repetition is one complete exercise movement; Set: A “set” is a group of consecutive reps; 1RM: one-repetition maximum

**Fig. 2:**
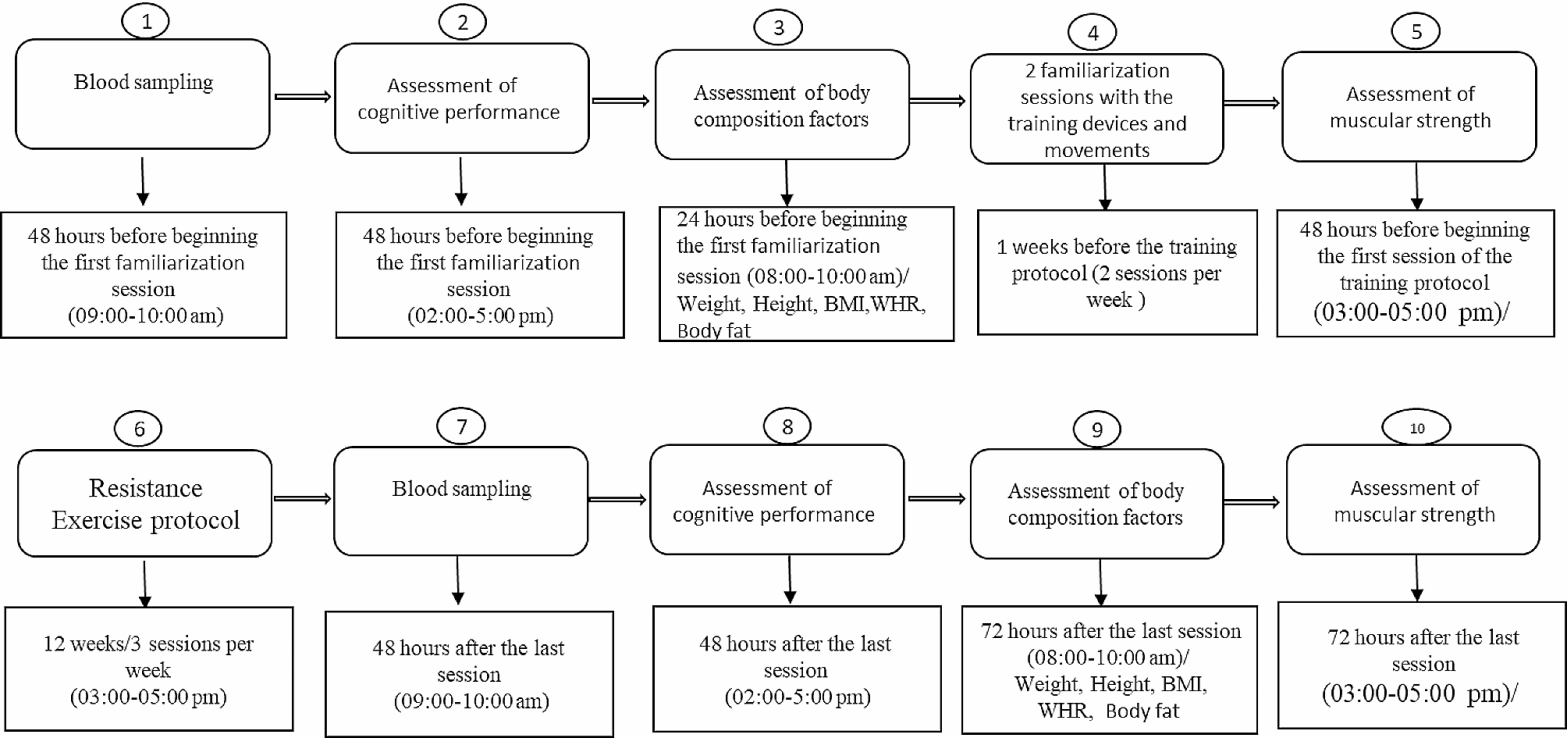
Diagram of the research plan

The experiment lasted for 13 weeks: 1 weeks for familiarization with the training devices and movements and 12 weeks for resistance training (MS + RT) or daily activity (for MS and HCON). Blood samples (8 cc) were collected from cubital veins after 12 h fasted state. In addition, body composition induces (height, age, weight, body fat percentage, body mass index (BMI), waist-to-hip ratio (WHR)), muscular strength (lower body and upper body) via five-repetition maximum (5RM) test [20] were measured and recorded. Pre- and post-test data were collected from all participants 24-48-72 h before and after the RT program as detailed in Fig. 2.

#### MMP-2, MMP-9, TIMP-1 and TIMP-2 and S100B serum levels measurements

Following blood collection, samples were centrifuged for all participants at a temperature of 4 °C and 3000 rpm for 10 min [9]. Thereafter, serum was separated and kept at − 80 °C until analysis. Serum levels of MMP-2, MMP-9, TIMP-1, TIMP-2 and S100B were measured by commercially available enzyme-linked immunosorbent assay kits [9, 18]: MMP-2, MMP-9, TIMP-1, TIMP-2, and S100B (Zellbio GmbH, Germany, with catalog number Cat. Nos: ZB-1973-H9648, ZB-1982- H9648, ZB-3003- H9648, ZB-11,218 C- H9648, and ZB-13,669 C- H9648, respectively). For all blood biochemistry measures the, intra-CV was < 10% and inter-CV was < 12%.

#### Assessment of cognitive performance

The Brief International Cognitive Assessment for MS (BICAMS) [21] was used to examine information processing speed and immediate verbal and visual recall. The BICAMS consists of the Symbol Digit Modalities Test (SDMT), California Verbal Learning Test-II (CVLT-II) and the Brief Visuospatial Memory Test- Revised (BVMT-R).

#### Resistance exercise training (RT) program

Based on the recommendations in RT for MS patients [22], the RT program included 12 weeks, 3 sessions/week, 60–80% 1RM for 60–90 min/session. The RT program consisted of three exercises for the lower extremity (leg press, lunges, and deadlift), three exercises for the upper extremity (bench press, wide grip lat pulldown and front dumbbell raise) (Table 1 outlines details).

#### Medication administration

The participants were monitored by neurologist and the drug was administered based on MS treatment in four groups of drugs (including Interferon beta, Glatiramer acetate, Dimethyl fumarate, Fingolimod). There was no difference between the drugs of all MS patients.

#### Statistical analysis

Distribution of data was assessed for normality using the Shapiro-Wilk test. A repeated-measure (RM) ANOVA 2 * 3 was used to evaluate the pre-post results of the three groups and to analyze the effect of time (pre–post) vs. the group (MS, MS + RT, and HCON). In addition, Bonferroni post hoc analysis was undertaken to compare the pairs. The effect size (ES) was also calculated as the change score divided by the SD of the change score to examine the magnitude of differences while controlling for the influence of the sample size, with 0.2 considered as a small ES, 0.5 as a moderate ES and > 0.8 as a large ES. The differences were accepted as significant if *P* ≤ 0.05. Data were expressed as mean ± SD.

### Results

#### Physiological indicators

As Table 2, for weight, Time x group interaction effects was not significant (F (2, 37) = 1.914, *p* = 0.112, Eta-squared = 0.112). Also, a 2 (Time) x 3 (Group) mixed-model ANOVA for weight data analysis revealed that the main effect for time was not significant (F (1, 37) = 0.370, *p* = 0.547, Eta-squared = 0.010). This means weight was not different between pre and post time, for three groups. In addition, no significant main effect for group was obtained (F (2, 37) = 0.568, *p* = 0.572, Eta-squared = 0.030). Thus, there was no overall difference in the weight between the three groups.

**Table 2:** Demographic characteristics of subjects with difference groups at baseline level and after intervention

Variables	Time	HCON(*n* = 15)	MS (*n* = 13)	MS + RT(*n* = 12)	Time effects p-value	Interaction effect (Time x Group) P-value
Age (years)		30/00 ± 6/0	35.15 ± 7.8	34.08 ± 8.9	-	-
Weight (kg)	Pre Post Pre-post P-value	68.89 ± 7.52 68.48 ± 7.14 *P* = 0.300	70.36 ± 12.99 70.86 ± 13.66 *P* = 0.093	65.37 ± 17.69 64.91 ± 17.19 *P* = 0.243	*P* = 0.547, Eta-squared = 0.010	*P* = 0.112 Eta-squared = 0.112
BMI (kg/m^2^)	Pre Post Pre-post P-value	24.10 ± 4.42 25.13 ± 1.85 *P* = 0.249	26.54 ± 4.23 26.71 ± 4.45 *P* = 0.118	24.40 ± 5.74 24.23 ± 5.50 *P* = 0.257	*P* = 0.391, Eta-squared = 0.020	*P* = 0.168 Eta-squared = 0.092
Body Fat (%)	Pre Post Pre-post P-value	29.89 ± 3.36 29.39 ± 3.42 *P* = 0.130	24.54 ± 7.14 24.90 ± 7.44 *P* < 0.05	22.00 ± 9.67 21.41 ± 9.10 *P* = 0.125	*P* = 0.149, Eta-squared = 0.056	*P* < 0.05 Eta-squared = 0.153
WHR (score)	Pre Post Pre-post P-value	0.78 ± 0.06 0.78 ± 0.05 *P* = 0.388	0.78 ± 0.06 0.78 ± 0.05 *P* = 0.532	0.78 ± 0.07 0.76 ± 0.06 *P* < 0.05	*P* < 0.05, Eta-squared = 0.125	*P* = 0.329 Eta-squared = 0.058

HCON: Health group; MS: Multiple Sclerosis; MS + RT: Multiple Sclerosis + resistance exercise training group; BMI: Body Mass Index; WHR: Waist-hip ratio

In addition, data analysis of BMI showed that Time x group interaction effects was not significant (F (2, 37) = 1.870, *p* = 0.168, Eta-squared = 0.092). Also, no significant main effect for time and group was obtained (F (1, 37) = 0.753, *p* = 0.391, Eta-squared = 0.020); (F (2, 37) = 1.009, *p* = 0.374, Eta-squared = 0.052), respectively) (Table 2).

However, a significant Time x Group interaction effects was also obtained for body fat percent (F (2, 37) = 3.349, *p* < 0.05, Eta-squared = 0.153). A comparison of means indicated that body fat percent was decreased after resistance training only in the MS + RT group, in comparison to other groups. In addition, the results of repeated measures ANOVA revealed that there was significant main effect for group (F (2, 37) = 4.590, *p* < 0.05, Eta-squared = 0.199). Pairwise comparison test showed that body fat percent in MS + RT group was significantly lower than HCON group (*p* < 0.05) (Table 2).

For WHR, Time x group interaction effects was not significant (F (2, 37) = 1.146, *p* = 0.329, Eta-squared = 0.058). However, a significant main effect for Time was obtained (F (1, 37) = 5.299, *p* = 0.027, Eta-squared = 0.125). Pairwise comparison test showed that WHR was significantly different only in MS + RT group (*p* < 0.05). This means WHR was decreased after resistance training. Moreover, the main effect for group was not significant (F (2, 37) = 0.176, *p* = 0.839, Eta-squared = 0.009). Thus, there was no overall difference between the three groups (Table 2).

#### Biological indexes

**Fig. 3:**
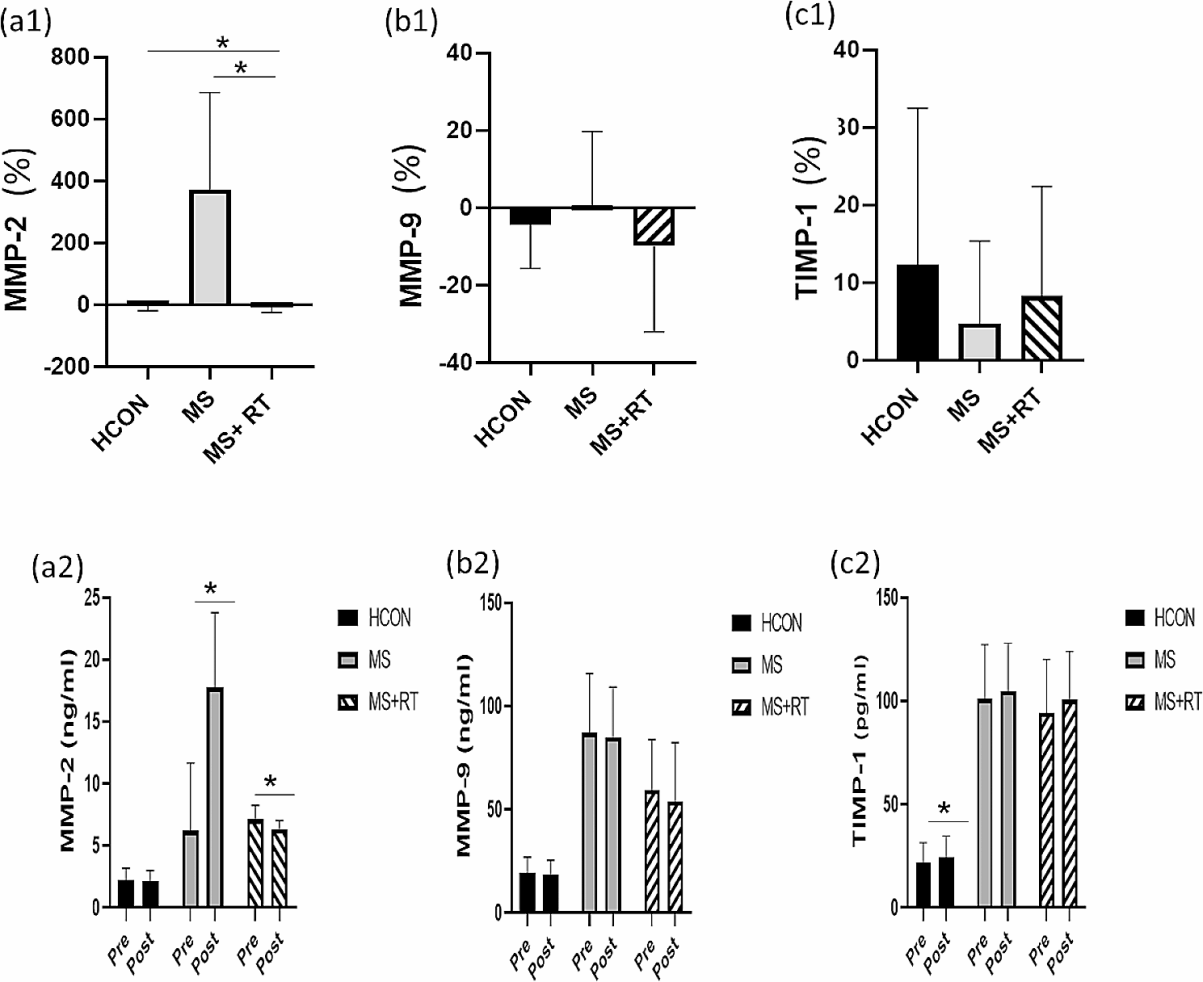
(**a1**) MMP-2; (**b1**) MMP-9; (**c1**) TIMP-1; indicated percentage changes in all groups; (**a2**) MMP-2; (**b2**) MMP-9; (**c2**) TIMP-1; indicated variables contents from pre to post- test in all groups; HCON group; MS group; MS + RT group. *Significant Time x Group interaction; effect *Significant pre-post different

For MMP2, a 2 (Time) x 3 (Group) mixed-model ANOVA revealed that a significant Time x Group interaction effect was observed (F (2, 37) = 28.377, *p* < 0.001, Eta-squared = 0.605, moderate ES). The comparison of means indicated that the MS + RT group had lower MMP2 levels after RT than baseline compared to the HCON and MS groups. There were significant pre–post differences for the time (F (1, 37) = 23.141, *p* < 0.001, Eta-Squared = 0.385, small ES). Where, MMP2 significantly increased in MS group (*p* < 0.001) and decreased in the MS + RT group (*p* < 0.05) (Table 3) (Fig. 3. (a1, 2)).

**Table 3:** The comparison of biological indexes in pre and post-test (Time effects) and between three groups (Time x Group interaction effects) (mean ± SD)

Variables	Time	HCON (*n* = 15) (mean ± SD)	MS (*n* = 13) (mean ± SD)	MS + RT(*n* = 12) (mean ± SD)	Time effects p-value	Interaction effect (Time x Group) P-value
MMP2 (ng/ml)	Pre Post Pre-post P value	2.23 ± 0.95 2.19 ± 0.88 *P* = 0.776	6.24 ± 5.42 17.75 ± 6.02 *P* < 0.001	7.11 ± 1.15 6.35 ± 0.68 *P* < 0.05	*P* < 0.001, Eta-squared = 0.385	$*P* < 0.001 Eta-squared = 0.605
TIMP2 (ng/ml)	Pre Pos Pre-post P value	0.69 ± 0.25 0.72 ± 0.23 *P* = 0.567	5.00 ± 1.49 4.92 ± 1.77 *P* = 0.632	6.10 ± 2.26 6.99 ± 2.31 *P* < 0.05	*P* < 0.05, Eta-squared = 0.110	*P* ≤ 0.01 Eta-squared = 0.225
MMP-9 (ng/ml)	Pre Post Pre-post P value	19.47 ± 7.60 18.45 ± 7.09 *P* = 0.067	87.18 ± 28.56 84.09 ± 24.26 *P* = 0.599	59.41 ± 24.35 54.07 ± 28.26 *P* = 0.108	*P* = 0.091, Eta-squared = 0.075	*P* = 0.560 Eta-squared = 0.031
TIMP-1 (pg/ml)	Pre Post Pre-post P value	21.93 ± 9.63 24.27 ± 10.50 *P* < 0.05	101.08 ± 26.06 104.75 ± 23.13 *P* = 0.231	94.36 ± 25.82 100.55 ± 23.32 *P* = 0.128	*P* < 0.05, Eta-squared:0.163	*P* = 0.580 Eta-squared = 0.029
MMP-2/TIMP-2 (Ratio)	Pre Post Pre-post P value	3.66 ± 1.80 3.33 ± 1.61 *P* = 0.261	1.20 ± 0.81 4.08 ± 2.57 *P* < 0.01	1.37 ± 0.68 1.02 ± 0.41 *P* < 0.05	*P* < 0.05, Eta-squared:0.153	*P* < 0.001 Eta-squared = 0.435
MMP-9/TIMP-1 (Ratio)	Pre Post Pre-post P value	0.97 ± 0.38 0.82 ± 0.30 *P* < 0.05	0.89 ± 0.31 0.82 ± 0.19 *P* = 0.254	0.67 ± 0.33 0.56 ± 0.33 *P* = 0.061	*P* < 0.01, Eta-squared:0.244	*P* = 0.565 Eta-squared = 0.030
S100B (ng/ml)	Pre Post Pre-post P value	0.1136 ± 0.0045 0.1135 ± 0.0042 *P* = 0842	0.1479 ± 0.0157 0.1485 ± 0.0179 *P* = 0.721	0.1420 ± 0.0096 0.1393 ± 0.0138 *P* = 0.332	*P* = 0.445 Eta-squared:0.016	*P* = 0.371 Eta-squared = 0.052

HCON: Health group; MS: Multiple Sclerosis; MS + RT: Multiple Sclerosis + resistance training group; MMP-2: Matrix metalloproteinases-2; TIMP-2: Tissue inhibitors of metalloproteinase-2; MMP-9: Matrix metalloproteinases-9; TIMP-1: Tissue inhibitors of metalloproteinase-9; MMP-2/TIMP-2 ratio: Matrix metalloproteinases-2/ Tissue inhibitors of metalloproteinase 2 ratio; MMP-9/TIMP-1 ratio: Matrix metalloproteinases-9/ Tissue inhibitors of metalloproteinase 1 ratio; S100B: S100 calcium-binding protein B

For MMP-9, no significant results were obtained for Time x Group interaction effects were also obtained (F (2, 37) = 0.589, *p* = 0.560, Eta-squared = 0.031, small ES). Main effect for Time was not observed (F (1, 37) = 3.007, *p* = 0.091, Eta-Squared = 0.075, small ES) (Table 3) (Fig. 3. (b1, 2)).

**Fig. 4:**
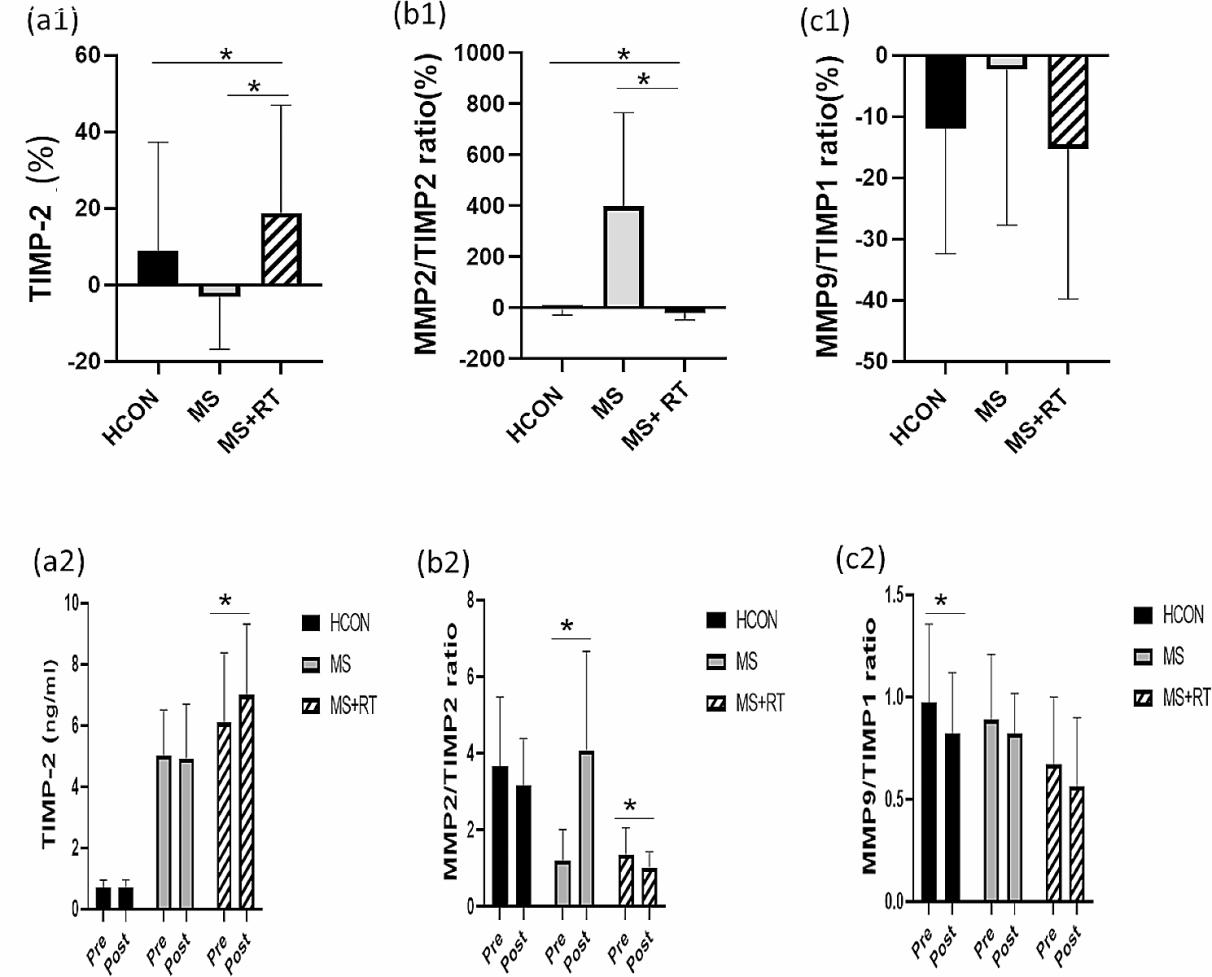
(**a1**) TIMP-2; (**b1**) MMP-2/ TIMP-2 ratio; (**c1**) MMP-9/TIMP-1 ratio; indicated percentage changes in all groups; and (**a2**) TIMP-2; (**b2**) MMP-2/ TIMP-2 ratio; (**c2**) MMP-9/TIMP-1 indicated variables contents from pre to post- test in all groups; HCON group; MS group; MS + RT group. *Significant Time x Group interaction; effect *Significant pre-post different

For TIMP1, no significant Time x Group interaction effects were observed (F (2, 37) = 0.553, *p* = 0.580, Eta-squared = 0.029, small ES). Although, there was significant pre–post differences for the time in the TIMP1 for all of groups (F (1, 37) = 7.218, *p* < 0.05, Eta-Squared = 0.163, small ES). The pairwise comparison test showed that the TIMP1 levels in the post-test were significantly higher than in the pre-test for the HCON group (*p* < 0.05) (Table 3) (Fig. 3. (c1, 2)). For TIMP2, a significant Time x Group interaction effect was obtained (F (2, 37) = 5.383, *p* < 0.01, Eta-squared = 0.225, small ES). In addition, there was main effect for Time (F (1, 37) = 4.592, *p* < 0.05, Eta-Squared = 0.110, small ES). The pairwise comparison test showed that the TIMP2 levels in the post-test significantly increased only in the MS + RT group (*p* < 0.05). Moreover, Comparison of means indicated that the MS + RT group had higher TIMP2 levels after RT rather than the pre-test in comparison to the HCON and MS groups (Table 3) (Fig. 4. (a1, 2)).

For MMP2/TIMP2, a 2 (Time) x 3 (Group) mixed-model of Repeated measures ANOVA revealed that a significant Time x Group interaction effect was also obtained (F (2, 37) = 14.232, *p* < 0.001, Eta-squared = 0.435, small ES). Comparison of means indicated that the MS + RT group had lower MMP2/TIMP2 levels after RT rather than the pre-test compared to the HCON and MS groups. In addition, there was a main effect for Time (F (1, 37) = 6.661, *p* < 0.05, Eta-Squared = 0.153, small ES). Where, MMP2/TIMP2 was increased in the MS group (*p* < 0.05), and it decreased in the MS + RT group (*p* < 0.05) (Table 3) (Fig. 4. (b1, 2)).

No significant Time x Group interaction effects was obtained for MMP-9/TIMP-1, F (2, 37) = 0.580, *p* = 0.565, Eta-squared = 0.030, small ES). Moreover, for MMP-9/TIMP-1, there was a main effect for Time (F (1, 37) = 11.926, *p* < 0.01, Eta-Squared = 0.244, small ES). Pairwise comparison test showed that, only for HCON group, the MMP-9/TIMP-1 ratio in the post-test was significantly lower than the pre-test (*p* < 0.05). In addition, the main effect for Group was not significant (F (2, 37) = 3.168, *p* = 0.054, Eta-squared = 0.146, small ES) (Table 3) (Fig. 4. (c1, 2)).

**Fig. 5:**
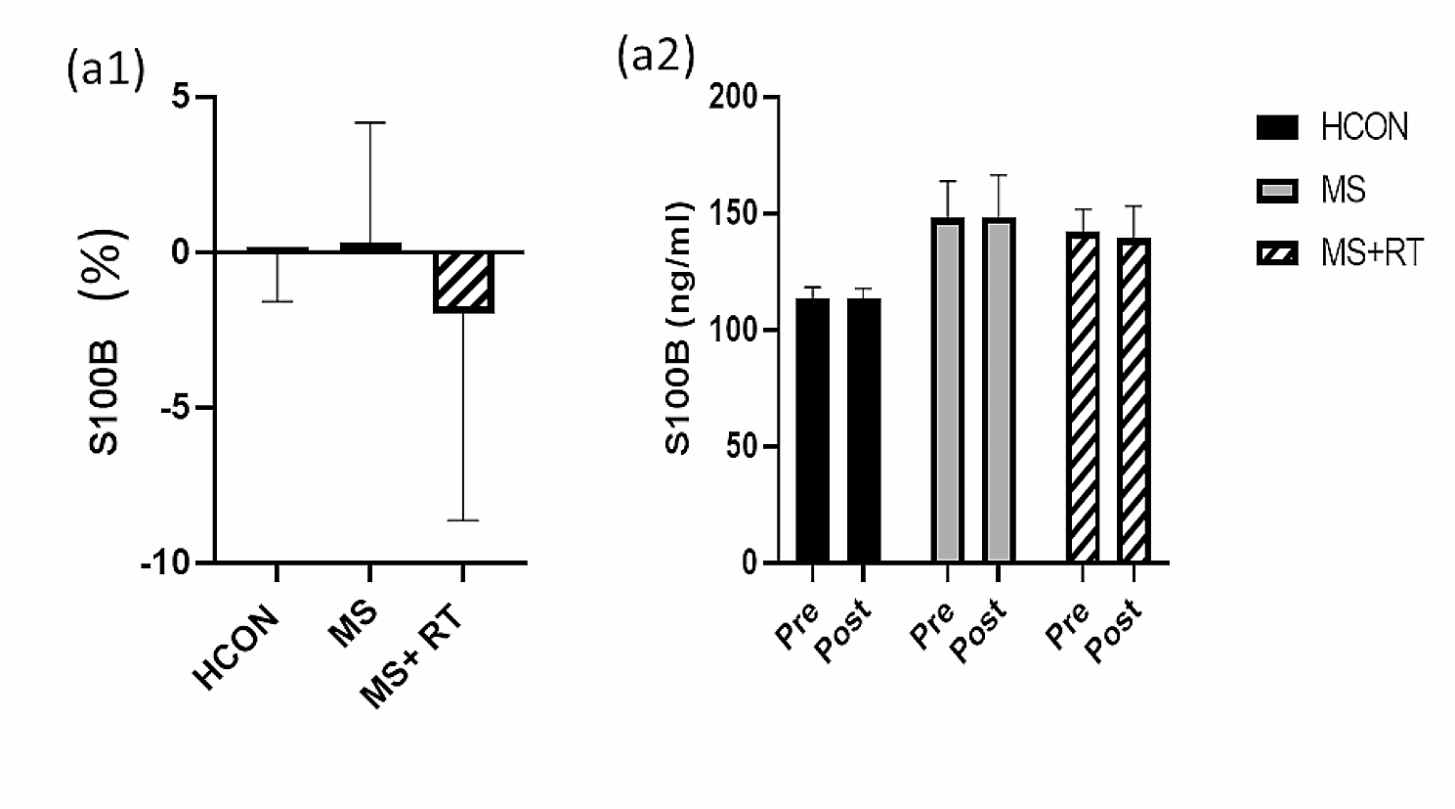
(**a1**) S100B; indicated percentage changes in all groups; and (**a2**) S100B; indicated variables contents from pre to post- test in all groups; HCON group; MS group; MS + RT group. *Significant Time x Group interaction effect; *Significant pre-post different

For S100B, no significant Time x Group interaction effects was obtained (F (2, 37) = 1.019, *p* = 0.371, Eta-squared = 0.052, small ES). And no significant results were obtained for any of conditions (Table 3) (Fig. 5. (a1, 2)).

#### Cognitive indexes

**Fig. 6:**
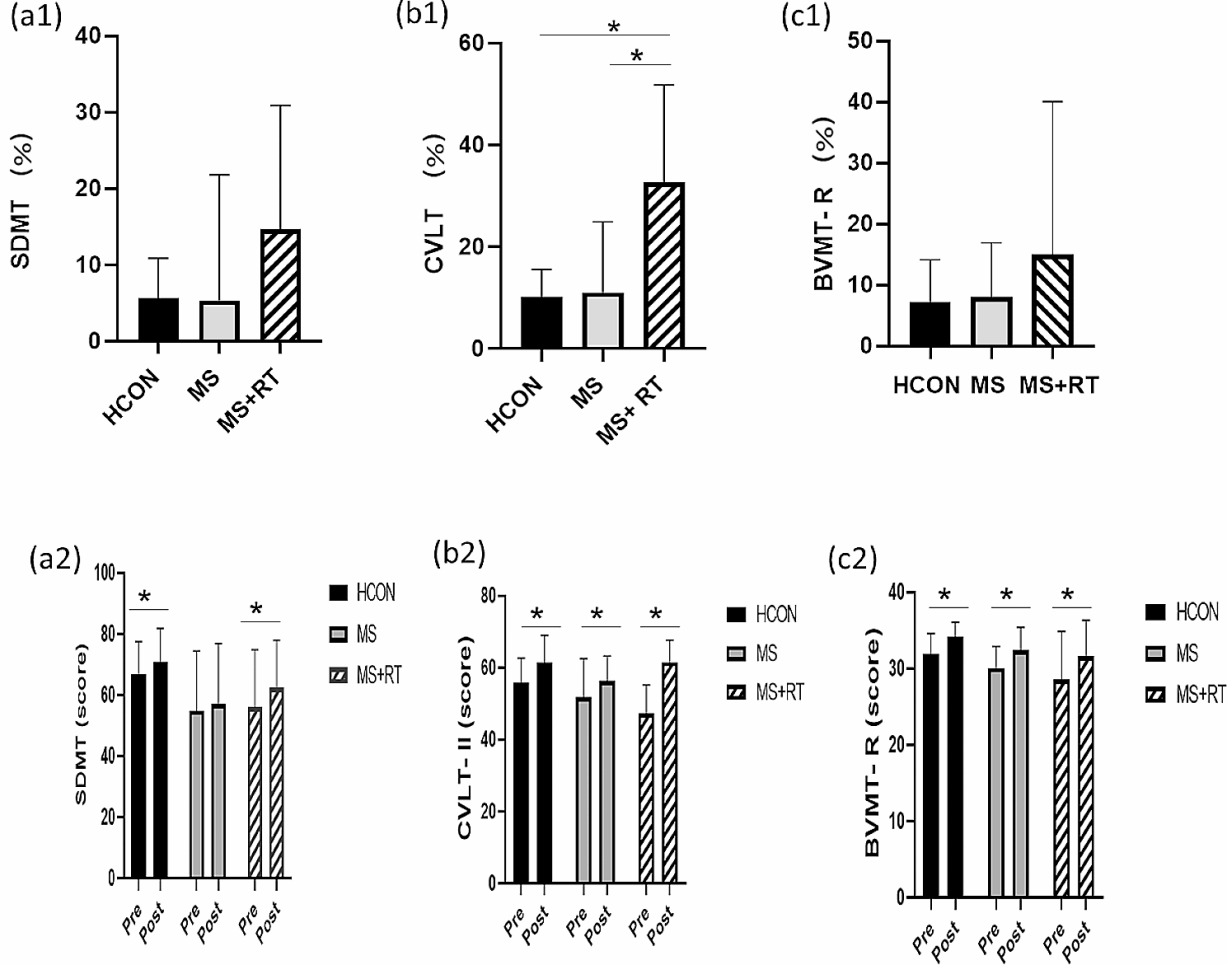
(**a1**) SDMT; (**b1**) CVLT-II; (**c1**) BVMT-R; indicated percentage changes scores in all groups; and (**a2**) SDMT; (**b2**) CVLT-II; (**c2**) BVMT-R; indicated scores from pre to post- test in all groups; HCON group; MS group; MS + RT group. *Significant Time x Group interaction; effect *Significant pre-post different

For SDMT, a non-significant Time x Group interaction effects were obtained (F (2, 37) = 1.023, *p* = 0.370, Eta-squared = 0.052, small ES). In addition, there was a main effect for Time (F (1, 37) = 12.933, *p* < 0.01, Eta-Squared = 0.259, small ES). The pairwise comparison test showed that the SDMT score in the post-test was significantly increased compared to the pre-test in HCON and MS + RT groups (*p* < 0.05) (Table 4) (Fig. 6. (a1, 2)).

**Table 4:** The comparison of cognitive indexes from pre- to post-test (Time effects) and between three groups (Time x Group interaction effects) (mean ± SD)

Variables	Time	HCON (*n* = 15) (mean ± SD)	MS (*n* = 13) (mean ± SD)	MS + RT (*n* = 12) (mean ± SD)	Time effects p-value	Interaction effects (Time x Group) P-value
SDMT (score)	Pre Post Pre-post P value	66.86 ± 10.61 70.60 ± 11.21 *P* < 0.001	54.76 ± 19.64 57.07 ± 19.70 *P* = 0.435	55.91 ± 18.88 62.33 ± 15.55 *P* < 0.01	*P* < 0.01, Eta-squared:0.259	*P* = 0.370 Eta-squared = 0.052
CVLT-II (score)	Pre Post Pre-post P value	55.80 ± 6.85 61.40 ± 7.56 *P* < 0.001	51.76 ± 10.70 56.30 ± 6.93 *P* < 0.05	47.25 ± 7.91 61.50 ± 6.12 *P* < 0.001	*P* < 0.001, Eta-squared:0.748	*P* < 0.001 Eta-squared = 0.445
BVMT- R(score)	Pre Post Pre-post P value	31.93 ± 2.65 34.13 ± 1.92 *P* < 0.01	30.07 ± 2.81 32.38 ± 2.98 *P* < 0.01	28.50 ± 6.36 31.58 ± 4.71 *P* < 0.05	*P* < 0.001, Eta-squared:0.442	*P* = 0.713 Eta-squared = 0.018

HCON: Health group; MS: Multiple Sclerosis; MS + RT: Multiple Sclerosis + resistance training group; SDMT: Symbol Digit Modalities Test; CVLT-II: California Verbal Learning Test-II; BVMT-R: Brief Visuospatial Memory Test- Revised

A significant Time x Group interaction effect was obtained for CVLT-II (F (2, 37) = 14.841, *p* < 0.001, Eta-squared = 0.445, small ES). The comparison of means indicated that the MS + RT group had a higher increase in CVLT-II scores in compare with other two groups. While, there was a solid main effect for Time (F (1, 37) = 109.574, *p* < 0.001, Eta-Squared = 0.748, moderate ES). The pairwise comparison test showed that CVLT-II scores in the post-test were significantly increased for all groups (*p* < 0.05) (Table 4) (Fig. 6. (b1, 2)).

Also, non-significant Time x Group interaction effects were obtained for BVMT-R (F (2, 37) = 0.342, *p* = 0.713, Eta-squared = 0.018, small ES). Where, there were significant pre–post differences for the time in the BVMT-R for all of groups (F (1, 37) = 126.967, *p* < 0.001, Eta-Squared = 0.442, small ES) (Table 4) (Fig. 6. (c1, 2)).

### Discussion

The purpose of current study was to investigate the effects of resistance training (RT) on serum biomarkers of BBB pathology and cognitive performance with MS women (MS-W). The results showed that RT significantly reduced the serum concentration of MMP-2 and MMP2/TIMP2 and increased the serum concentration of TIMP2 in MS + RT compared to the MS and HCON groups. However, Changes in MMP-9, TIMP-1, s100B and MMP-9/TIMP-9 in the MS + RT group were non-significant compared to the MS and HCON groups. In addition, regarding cognitive function, RT led to a significant improvement in verbal memory in MS + RT over 12 weeks.

In the current study, baseline serum levels of MMP-2 and MMP-9 were significantly higher in the MS and MS + RT groups than in the HCON group. Physiological studies suggest that MMPs overexpression in MS induces infiltration of CD4 + Th1 cells and macrophages across the BBB and myelin and/or axonal damage and is associated with disability and disease severity [10, 12, 13, 23]. In accordance with our results, clinical studies demonstrated a significant elevation in MMP-9 and MMP-2 serum levels in the RRMS, primary progressive form (PP-MS) and secondary progressive MS (SPMS) groups compared to healthy controls [11, 12, 24]. In a study on RR-MS patients, the increase in MMP-9 serum levels was associated with BBB disruption [11]. Also, elevated levels of MMP-9 in both attack and remission periods in patients with RR-MS have been observed [24].

Our results suggest that RT led to a decrease in serum levels of MMP-2 while MMP-9 unchanged in the MS + RT group compared to the HCON and MS groups. This findings showed the role of resistance training to modify BBB biomarkers although there is limitation in the research studies in this area. Here, a pervious study [9] mentioned findings congruous with our study. This study, performed a three-week high-intensity aerobic exercise, indicated significantly reduction in MMP-2 but not MMP-9 in MS patients. Conversely, Deckx et al. reported a decrease in MMP-9 production at the end of a 12-week exercise (combined endurance and RT) in MS patients [19]. Despite limited studies in MS patients, MMP-2 and MMP-9 have been studied after different exercise training programs in other chronic diseases [25–28]. For example, Kadoglu et al. demonstrated a decrease in serum level of MMP-9 and no changes in MMP-2 in overweight patients with type 2 diabetes (T2DM) [25]. However, our study did not show a significant change for serum level of MMP-9 after 12 weeks of RT. It can be speculated that differences in training duration period, training intensity and pathology of different diseases might explain the inconsistencies in findings. Therefore, improving BBB integrity by reducing serum MMP-2 concentrations, a primary outcome of this study appears to be an integral component for reducing MS-specific symptoms and disease progression.

Our observations demonstrated that the serum level of TIMP-1 and TIMP-2 were higher in MS-W in the MS and MS + RT groups than in the HCON group. Consistent with our study, elevated levels of TIMP-1 and TIMP-2 in the serum of MS patients have been observed in different studies [7]. TIMPs inhibit the proteolytic activity of MMPs [29]. As part of a complex system of regulation, TIMP-1 is a tissue inhibitor of MMP-9 [7]. Besides, TIMP-2 is a well-known MMP-2 inhibitor that are present in the ECM in a soluble form [7]. When considering the role of resistance training, we observed an improvement in TIMP1and TIMP-2 levels in the MS + RT group compared to other groups. It has been previously demonstrated an increase in serum levels of TIMP-1 and TIMP-2 in chronic diseases after exercise training [25, 27, 30]. To the best of our knowledge, the possible effect of exercise intervention on serum TIMP-1 and TIMP-2 concentration as well as MMP/TIMP homeostasis in MS patients remains unknown.

Measuring MMPs/TIMPs ratio is important because a critical balance between circulating MMPs and TIMPs must exist, and MMPs/TIMPs ratio may be useable indexes of net MMPs activities [31]. In our study, MMP-9/TIMP-1 and MMP2/TIMP2 ratio were assessed. Similar to previous study [12] findings, we determined that values for both ratios was observed higher in MS-W than healthy people. It has been have suggested that the MMPs/TIMPs increase is associated with BBB disruption and the number of lesions increase in MS patients [11, 13, 32]. Liuzzi et al. reported a significant inverse correlation between MMP-9 and TIMP-1 with BBB disruption in RR-MS [13]. Furthermore, serum levels of MMP-2 and MMP-2/TIMP-2 ratio increase have previously caused enhanced BBB permeability and degradation of basement membrane via tight junction protein phosphorylation and are associated with disability and disease severity [12, 32]. We showed the MMP-2/TIMP-2 ratio decrease in the MS + RT group whereas it increased in MS groups. This change in the MMP-2/TIMP-2 ratio was due to the decrease in MMP-2 and the increase in TIMP-2 in the RT-MS group. It should be remarked that we could not find studies to measure MMP/TIMP ratio in MS patients after exercise training.

In addition, when considering this blood-brain barrier disorder due to an elevated serum MMP-2/TIMP-2 ratio [12, 32], exercise training can maintain blood-brain barrier integrity [33], and the anti-inflammatory response promoted by exercise training reduces TNF-α and IL-6 [34]. Razi et al. recently reported that aerobic training maintained BBB integrity through reducing astrogliosis and Ang-1 and reversing EAE-induced TJ protein down-regulation, and that a six-week aerobic training program has provided a protective role to neurons via attenuation of neuronal apoptosis [33]. However, we were not able to detect differences in MMP-9/TIMP-1 following 12 weeks of exercise. Kadoglou et al. reported a decrease in MMP-9/TIMP-1 ratio after exercise training program in T2DM [25]. Although the type and duration of exercise are important components in exercise training programs, our results showed resistance training effect in MS patients. Further studies need to clarify the reasons for the difference results in MS patients.

It has been reported that people with MS higher serum level of S100B compared to healthy control [35, 36]; which was also observed in the current study. S100B level in the serum has been used as a marker for BBB disintegration and brain damage [3, 18]. Studies suggest that the inhibition of the S100B may be protective against both demyelination and consequent axonal injury [36]. We did not detect any significant differences following 12 weeks of RT in the S100B serum level as an index of BBB permeability; however, adjusted plots of these analyses and univariate tests indicated a trend towards a decline in MS + RT and an elevation for MS Together, these results suggest that resistance training may be effective on this factor. Contrary to our results, other studies that used HIIT and endurance training reported significant reductions in S100B levels (in MS and Parkinson patients, respectively) [18, 37]. Probably, the nature of training and the type of activity (resistance training vs. aerobic training) and training intensity (HIIT) play a major role in the activation of different signals; however, Chupel et al. observed that combined exercise for 14 weeks preserves BBB integrity and reduces chronic inflammation in elderly women with no reduction in S100B [38].

Studies have reported improved cognitive functions in MS patients following an aerobic training program [9, 39]. In our study, data analysis showed the significant time effects for SDMT in healthy and MS + RT groups, and for CVLT-II, and BVMT-R in all three groups. Excluding verbal learning (CVLT-II) in the MS + RT group, the differences were insignificant regarding Time x Group interaction. These findings are in-line with the results of an RCT study suggesting that HIT improved verbal memory in individuals with MS in-patient rehabilitation [9]. Changes in CVLT-II scores were strongly associated with changes of the hippocampus [39]. Conversely, Oken et al. reported that MS patients who participated in a 6-month yoga programme showed no relative improvement in cognitive function in either intervention groups [40]. One pilot study conducted by Kierkegaard et al. investigated the effect of 12 weeks of high-intensity RT on the cognitive performance of MS patients, and reported the positive effects of training on information SDMT [41]. However, our findings are limited due to the lack of a clearly in-defined level of cognitive impairments at baseline.

Our results showed that 12 weeks of resistance training decreased body fat percentage (BF %) and WHR in women with MS. It has been shown that Decreasing the BF % with resistance exercises leads to the reduction of inflammatory factors (IFNγ and TNF -α) which are factors involved in the pathophysiology of MS [42, 43]. The results of our study are consistent with previous studies that resistance training can reduce fat percentage and coronary artery disease risk factors in women with MS [44, 45]. However, recent studies have shown that resistance exercises can positively affect strength, balance, physical functioning, and self-care strategies, all of which improved their sleeping, emotion regulation, depression, and participation in social activities [43, 46, 47]. The main limitation of the study was the date when COVID-19 was occurred. Therefore, the small groups of MS patients participated in our study. Another limitation was the non-controlled diet which could influence on neuroinflammation. One of the strengths of current study was that resistance training supervized for safety by an experienced staff. Also, compared to the endurance exercise, resistance training in heat sensitive patients less frequently cause symptom exacerbations due to increased body temperature.

### Conclusion

The present results suggest that resistance training in the form of moderate-intensity resistance exercise training may modify and manage BBB pathology markers of MS, although the role of S100B, MMP-9, TIMP-1, and MMP-9/TIMP-1 ratio in MS remains unclear when considering exercise training. Since MMP-2 has been described to be mediated inflammatory processes, exercise-induced MMP-2 and TIMP-2 changes support the hypothesis that regular exercise has mediated anti-inflammatory properties. Future research with considering exercise type, intensity, duration and effects on BBB permeability and cognitive function in MS patients.

## Data Availability

The data and information are available with the correspondence author if requested.

## Abbreviations

1RM: One-repetition maximum
BBB: Blood brain barrier
BICAMS: Brief International Cognitive Assessment for MS
BVMT-R: Brief Visuospatial Memory Test- Revised
CVLT-II: California Verbal Learning Test-II
EAE: Experimental autoimmune encephalomyelitis
EDSS: Expanded Disability Status Scale
HCON: Healthy control group
MMP-2: Matrix metalloproteinase-2
MMP-9: Matrix metallopeptidase-9 (MMP-9)
MS: Multiple sclerosis
RT: Resistance exercise training
RRMS: Patients with relapsing-remitting MS
S100B: S100 calcium-binding protein B
SDMT: Symbol Digit Modalities Test
TIMP-1: Tissue metalloproteinase inhibitors-1
TIMP-2: Tissue metalloproteinase inhibitors-2
MS-W: MS Women
MS + RT: MS + resistance exercise training
